# Differential Regulation of Growth-Promoting Signalling Pathways by E-Cadherin

**DOI:** 10.1371/journal.pone.0013621

**Published:** 2010-10-26

**Authors:** Nikolaos T. Georgopoulos, Lisa A. Kirkwood, Dawn C. Walker, Jennifer Southgate

**Affiliations:** 1 Jack Birch Unit for Molecular Carcinogenesis, Department of Biology, University of York, York, United Kingdom; 2 Department of Computer Science, Kroto Research Institute, University of Sheffield, Sheffield, United Kingdom; The Beatson Institute for Cancer Research, United Kingdom

## Abstract

**Background:**

Despite the well-documented association between loss of E-cadherin and carcinogenesis, as well as the link between restoration of its expression and suppression of proliferation in carcinoma cells, the ability of E-cadherin to modulate growth-promoting cell signalling in normal epithelial cells is less well understood and frequently contradictory. The potential for E-cadherin to co-ordinate different proliferation-associated signalling pathways has yet to be fully explored.

**Methodology/Principal Findings:**

Using a normal human urothelial (NHU) cell culture system and following a calcium-switch approach, we demonstrate that the stability of NHU cell-cell contacts differentially regulates the Epidermal Growth Factor Receptor (EGFR)/Extracellular Signal-Regulated Kinase (ERK) and Phosphatidylinositol 3-Kinase (PI3-K)/AKT pathways. We show that stable cell contacts down-modulate the EGFR/ERK pathway, whilst inducing PI3-K/AKT activity, which transiently enhances cell growth at low density. Functional inactivation of E-cadherin interferes with the capacity of NHU cells to form stable calcium-mediated contacts, attenuates E-cadherin-mediated PI3-K/AKT induction and enhances NHU cell proliferation by allowing de-repression of the EGFR/ERK pathway and constitutive activation of β-catenin-TCF signalling.

**Conclusions/Significance:**

Our findings provide evidence that E-cadherin can differentially and concurrently regulate specific growth-related signalling pathways in a context-specific fashion, with direct, functional consequences for cell proliferation and population growth. Our observations not only reveal a novel, complex role for E-cadherin in normal epithelial cell homeostasis and tissue regeneration, but also provide the basis for a more complete understanding of the consequences of E-cadherin loss on malignant transformation.

## Introduction

E-cadherin is a member of the cadherin family of transmembrane glycoproteins that mediates formation of adherens junctions in epithelial cells [1]. Formation of calcium-dependent “zipper-like” homophilic interactions between the extracellular domains of E-cadherin on adjacent cells facilitates epithelial cell-cell contact and adhesion [2]. These interactions are stabilised by the association of the cytoplasmic tail of E-cadherin with the α, β and γ catenins, which anchor E-cadherin to the cytoskeleton [3].

In epithelial tissues, E-cadherin functions not only to stabilise homotypic cell interactions, but also to modulate extracellular growth-associated signals, thus providing a homeostatic mechanism to link cell-cell contact/adhesion with cellular proliferation and tissue growth. For instance, E-cadherin interacts with β-catenin, a multifunctional protein that regulates cell growth by acting as a transcription factor in Wnt signalling [4]. The association between E-cadherin and β-catenin not only stabilises the adherens complex itself, but also provides a mechanism by which E-cadherin can sequester β-catenin to the cell membrane and impede β- catenin/TCF-mediated transcription [5], [6]. Although the cytoplasmic tail of E-cadherin lacks enzymatic activity, it can regulate growth-promoting cell signalling, such as the Phosphatidylinositol 3-Kinase (PI3-K)/AKT and Extracellular Signal-Regulated Kinase (ERK) pathways, by influencing the activation of receptor tyrosine kinases (RTKs). Some studies have reported positive regulation of Epidermal Growth Factor Receptor (EGFR) downstream signalling through ERK [7], [8] and PI3-K/AKT [9] following calcium-mediated formation of E-cadherin intercellular contacts in monocultures of normal keratinocytes and cancer cell lines, respectively. Yet, using the same calcium-switch approach, others have demonstrated that E-cadherin-mediated cell-cell adhesion can inhibit the ligand-dependent activation of diverse RTKs, including EGFR [10], [11]. Although these studies imply a critical, context-specific role for E-cadherin-dependent cell-cell contacts in modulating growth-promoting intracellular signalling pathways, many of the described findings are contradictory. Equally importantly, it remains unknown whether E-cadherin can simultaneously regulate these pathways and/or how the influence of E-cadherin on these signalling cues dictates epithelial cell behaviour, particularly within the tissue context.

We have previously developed a culture system for normal human urothelial (NHU) cells [12]. In low calcium (0.09 mM), serum-free culture medium, NHU cells adopt a highly proliferative, regenerative phenotype which is driven via the ERK, mitogen-activated protein kinase (MAPK) cascade downstream of an EGFR-activated autocrine pathway, with amphiregulin and HB-EGF identified as the key endogenous ligands [13]. A switch to physiological (2 mM) calcium induces stratification and the formation of adherens and tight junctional complexes, but does not induce urothelial cytodifferentiation [14], although the cells retain the capacity to differentiate [15] and to form a functional barrier epithelium [16].

To aid interpretation of the cellular basis of tissue regulatory mechanisms, we have previously developed the “Epitheliome”, an agent-based computational model of epithelial cell behaviour [17], [18], [19]. By incorporating rule sets to describe concepts of calcium-dependent bonding and a G1 checkpoint operated by contact inhibition, we have shown good qualitative agreement between in virtuo simulations and experimentally-acquired growth kinetics for NHU cell cultures. In low (0.09 mM) exogenous calcium concentrations, both real and simulated cell cultures display higher growth rates and achieved greater final cell densities than equivalent cultures grown in physiological (2 mM) calcium, where the tendency towards colony formation results in early exit from cell cycle due to contact inhibition [17]. The single discrepancy was that low-density NHU cell cultures in 2 mM calcium conditions consistently showed a higher growth rate compared to cultures in 0.09 mM calcium, which was not replicated in the simulated system [17]. This has been examined computationally by exploring growth signals augmented through E-cadherin-dependent cell-cell contacts [20]. These observations predict a more complex interplay between signalling mechanisms for the regulation of cellular growth at different population densities.

The aim of our present study was to identify the growth-promoting pathways that underpin the above observations and contribute to the regulation of epithelial tissue homeostasis and regeneration. The urothelium, which is able to switch from a mitotically quiescent to a highly-proliferative regenerative tissue in response to damage, represents an ideal model system. Using a functional inactivation approach, we demonstrate that E-cadherin interactions simultaneously regulate multiple growth-promoting signalling pathways and that the pattern of activation of these pathways accounts for the cell-growth differences observed. Our findings not only reveal a complex role for E-cadherin in regulating growth signals in normal epithelial cells, but also provide further insight into the functional consequences of E-cadherin loss in neoplastic disease.

## Results

### The stability of NHU cell-cell contacts differentially regulates the EGFR/ERK and PI3-K/AKT signalling pathways

**Figure 1:**
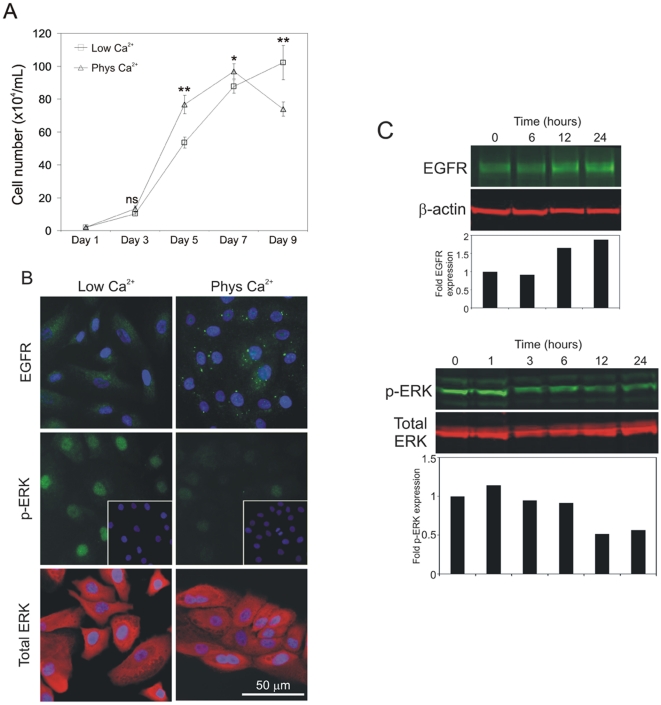
Physiological calcium causes a transient increase in proliferation in low density NHU cultures which is independent of EGFR/ERK signaling. (A) NHU cells were seeded in 6-well plates at 2×10^4^/well in medium containing 0.09 mM (Low Ca^2+^) or 2.0 mM (Phys Ca^2+^) extracellular [Ca^2+^]. Cell growth was determined by cell counting for a total period of 9 days. Each data point represents the mean (±S.E.M.) of 3 replicates (n = 3) and results are representative of at least three independent experiments. ns, non-significant; *, P<0.05; **, P<0.01. (B) NHU cells were cultured as above and expression of EGFR, phospho-ERK (pERK) and total ERK was assessed by immunofluorescence microscopy using rabbit (EGFR and p-ERK) and mouse (total ERK) antibodies, followed by goat antisera conjugated with Alexa Fluor 488 (green) or 594 (red). Cell nuclei were visualised using Hoechst 33258 (blue). (C) NHU cells were seeded in standard culture medium (0.09 mM calcium) and left to attach overnight. Calcium concentration was then increased to 2 mM and protein lysates were prepared at the indicated time-points for gel-electrophoresis and immunoblotting. Expression of EGFR, phospho-ERK (pERK), total ERK and β-actin was determined using mouse antibodies (total ERK/β-actin) and rabbit antisera (EGFR/pERK), followed by goat anti-mouse antibody conjugated with Alexa Fluor 680 (red) or goat anti-rabbit antibody conjugated with IRDye 800 (green). Densitometry was performed to determine fold induction of EGFR and pERK expression following normalisation against β-actin and total ERK, respectively. Bar graphs represent results relative to the expression level of the initial time point (0 hours).

Growth curves were constructed from cell counts of NHU cell cultures maintained in low (0.09 mM) and near physiological (2 mM) calcium concentrations. In all cases, cultures were maintained in the presence of saturating concentrations of exogenous EGF in order to limit any confounding effects from autocrine/juxtacrine EGFR activation [13]. NHU cell cultures maintained in 2 mM calcium exhibited higher growth rates during exponential growth phase than cultures grown in 0.09 mM calcium conditions. By contrast, cultures maintained in 0.09 mM calcium grew more slowly, but attained a higher ultimate cell density (Figure 1A).

As EGFR/ERK signalling is critical in NHU cell proliferation [13], we used immunofluorescence microscopy to examine the expression of EGFR and its downstream signalling effector, ERK, 24 hours following a switch from 0.09 mM to 2 mM calcium. The calcium switch resulted in a major down-regulation of nuclear phospho-ERK, despite the total amount of ERK remaining unchanged (Figure 1B). Along with an increase in the overall expression level of EGFR, we observed a change in localisation from diffuse cytoplasmic to intense, punctate labelling in cytoplasmic and perinuclear areas (Figure 1B). Immunoblotting studies, carried out to monitor EGFR and phospho-ERK over a 24-hour time-course, confirmed the increase in EGFR expression, which occurred within 12 hours of treatment, as well as the decrease in phospho-ERK as early as 3 hours following the calcium-switch (Figure 1C).

**Figure 2:**
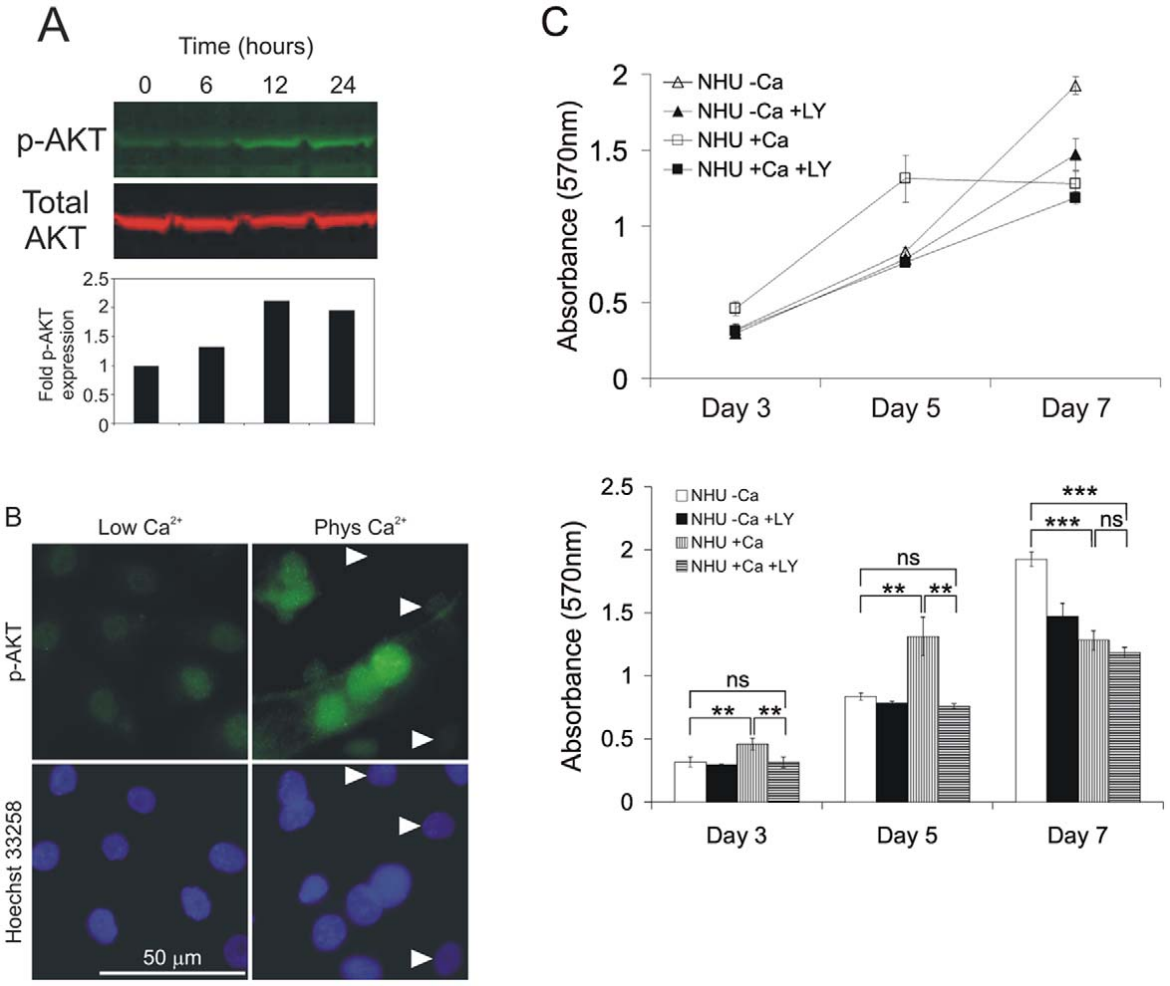
Calcium-induced increase in proliferation in low-density NHU cultures occurs via activation of the PI3-K/AKT pathway. (A) NHU cells were seeded in medium containing low [Ca^2+^] (0.09 mM) and left to attach overnight. Calcium concentration was then increased to 2 mM and protein lysates were prepared at the indicated time-points for immunoblotting. Expression of phospho-AKT (green) and total AKT (red) was determined using rabbit and mouse antibodies, respectively, followed by fluorochrome-conjugated secondary antisera as in Figure 1C. Immunolabelling was visualised and densitometry to determine fold induction of p-AKT expression with respect to total AKT was performed as in Figure 1C. (B) NHU cells were cultured in medium containing 0.09 mM (Low Ca^2+^) or 2.0 mM (Phys Ca^2+^) [Ca^2+^]. Expression of phospho-AKT (p-AKT) was determined by immunofluorescence microscopy using anti-p-AKT rabbit antibody followed by goat anti-rabbit antibody conjugated with Alexa Fluor 488 (green). Cell nuclei were visualised using Hoechst 33258 (blue). (C) NHU cells were seeded into 96-well plates and cultured for a period of 7 days in medium containing either low (-Ca) or physiological (+Ca) calcium levels, in the presence or absence of 5 µM of the PI3-K inhibitor LY294002. Proliferation was determined on the basis of cell biomass using the MTT assay. Data points represent mean absorbance values for 6 replicate wells (±S.E.M.). Results for all data points are also presented in the form of bar graphs (lower panel) for the purpose of statistical analysis. ns, non-significant; **, P<0.01; ***, P<0.001.

The reduction in phospho-ERK activity indicated that formation of calcium-mediated, ‘stable’ intercellular contacts did not result in increased EGFR/ERK signalling and suggested that this pathway is unlikely to be responsible for the increased proliferation rate in low-density cultures. As activation of the PI3-K/AKT pathway has been previously implicated in cell contact-dependent proliferation [9], [21], the possibility of an induction of AKT activity was investigated. Immunoblotting studies revealed the emergence of phosphorylated AKT on Ser-473, which occurred within 12 hours of switching to 2 mM calcium (Figure 2A); by contrast no phospho-AKT on Tyr-308 was observed (not shown). The findings were confirmed by immunofluorescence microscopy, which showed phospho-AKT to be nucleus-localised (Figure 2B). Only cells making direct contacts with neighbouring cells showed intense activation of AKT, whilst lone cells showed little, if any, detectable phospho-AKT (Figure 2B, right top panel, denoted by arrows). This implied that it was the formation of cell-cell contacts, rather than addition of calcium alone, which was critical for AKT activation. To corroborate direct functional involvement of the PI3-K/AKT pathway in the enhancement of growth rates, population growth was assessed for NHU cell cultures grown in 0.09 mM or 2 mM calcium in the presence or absence of the specific PI3-K inhibitor LY294002 over a 7 day time-course (Figure 2C). In confirmation of the kinetic data shown above (Figure 1A), NHU cells grown in 2 mM calcium showed higher proliferation rates during exponential growth, whereas cultures grown in 0.09 mM calcium achieved a higher density by day 7. The increased proliferation rate, characteristic of cultures grown in 2 mM calcium, was completely abolished by LY294002 treatment, whereas LY294002 had little effect on the exponential growth rate of cultures grown in low calcium medium (Figure 2C). These results indicate that stable, calcium-mediated, cell-cell interactions enhanced proliferation by activation of the PI3-K/AKT pathway in sub-confluent cultures. By contrast, the enhanced cell-cell contacts produced in physiological calcium conditions appeared to promote earlier exit from the cell cycle (as indicated by the lower growth rates at later time points), presumably due to cell contact inhibition.

### Expression of a dominant-negative E-cadherin mutant interferes with expression and localisation of endogenous E-cadherin and catenins

**Figure 3:**
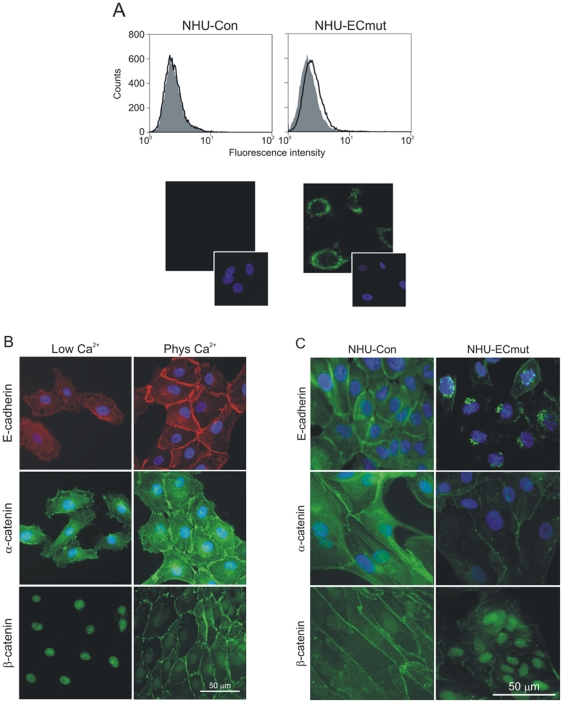
Effect of dominant-negative mutant E-cadherin on expression and localisation of endogenous E-cadherin and catenins in NHU cells. (A) NHU cells expressing the H-2K^d^-E-cad mutant (NHU-ECmut) and their isogenic controls (NHU-Con) were established by retrovirus transduction and expression of mutant E-cadherin was assessed by flow cytometry and immunofluorescence microscopy. For flow cytometry (upper panels), expression was analysed using FITC-conjugated anti-H-2K^d^ antibody (open histograms) alongside irrelevant isotype-matched control antibody (filled histograms). Histograms represent log_10_ fluorescence intensity in the FL-1 channel. For immunofluorescence microscopy (lower panels), expression was detected using anti-H-2K^d^ antibody followed by goat anti-mouse antibody conjugated with Alexa Fluor 488 (green). (B) Following initial seeding, NHU cells were cultured in medium containing low (0.09 mM) or physiological (2.0 mM) Ca^2+^ concentrations for 24 hours before expression of E-cadherin, α-catenin and β-catenin was assessed by microscopy using mouse (E-cadherin) and rabbit (catenins) antibodies, followed by goat antisera conjugated with Alexa Fluor 488 (green) or 594 (red). (C) NHU-Con and NHU-ECmut cells were cultured in medium containing physiological [Ca^2+^] and expression of E-cadherin, α-catenin and β-catenin was determined by labelling using primary antibodies above, followed by Alexa Fluor 488-conjugated antibody (green). In all immunofluorescence microscopy experiments, cell nuclei were visualised by labelling with Hoechst 33258 (blue).

To examine further the importance of cell-cell contacts in regulating proliferation, the effect of modifying adherens junctions was examined. Due to the critical role of E-cadherin in the formation of cell-cell contacts and its potential involvement in the regulation of such signalling pathways, we performed functional inactivation of E-cadherin in NHU cells by transduction with a retrovirus expressing the dominant-negative E-cadherin construct H-2K^d^-E-cad [22], [23]. The construct encodes a chimeric protein comprising the extracellular domain of H-2K^d^ class I and the transmembrane and intracellular domains of E-cadherin. Recombinant retrovirus expressing the E-cadherin mutant was used to infect NHU cells alongside a control virus, giving rise to isogenic sub-lines NHU-ECmut and NHU-Con, respectively. As our optimised retrovirus method for stable NHU cell transduction is >90% efficient [24], NHU sub-lines were derived as non-clonal populations, thus negating integration artefacts. By flow cytometry detection, the expression of surface H-2K^d^-E-cad on NHU-ECmut cells was very low (Figure 3A, upper panels), but immunofluorescence microscopy showed substantial intracellular expression, with the H-2K^d^-E-cad protein localising mainly to perinuclear areas (Figure 3A, lower panels).

In low calcium medium, NHU cells showed moderate, diffuse cytoplasmic E-cadherin expression, which became intense and localised to intercellular borders within hours of switching to physiological calcium (Figure 3B). Associated with the calcium-mediated stabilisation of E-cadherin, the major cytoplasmic-domain partners of E-cadherin were also relocated to different intracellular compartments. Thus, α-catenin showed a change from diffuse cytoplasmic to intense plasma membrane and cytoskeletal filament localisation, whereas β-catenin relocalised from the nucleus to cell-cell contact points (Figure 3B). Expression of the dominant-negative E-cadherin construct H-2K^d^-E-cad caused a dramatic change in the pattern of endogenous E-cadherin localisation. In the retrovirus-transduced NHU-ECmut cells maintained in 2 mM calcium, E-cadherin was detected in perinuclear, Golgi/late-endosome compartments (compare left and right panels, Figure 3C), synonymous with the perinuclear localisation of the H-2K^d^-E-cad chimera (lower panels, Figure 3A). Associated with the loss of surface E-cadherin in NHU-ECmut cells, there was down-regulation of α-catenin and increased expression of nuclear β-catenin (Figure 3C). These experiments confirmed that the H-2K^d^-E-cad mutant expressed in NHU cells was functional, as it efficiently incapacitated endogenous E-cadherin by blocking localisation to sites of cell-cell contact and preventing recruitment of α- and β-catenin.

### Functional inactivation of E-cadherin enhances NHU cell proliferation and reveals a pattern of E-cadherin-mediated differential regulation of EGFR/ERK, PI3-K/AKT and β-catenin/TCF signalling

**Figure 4:**
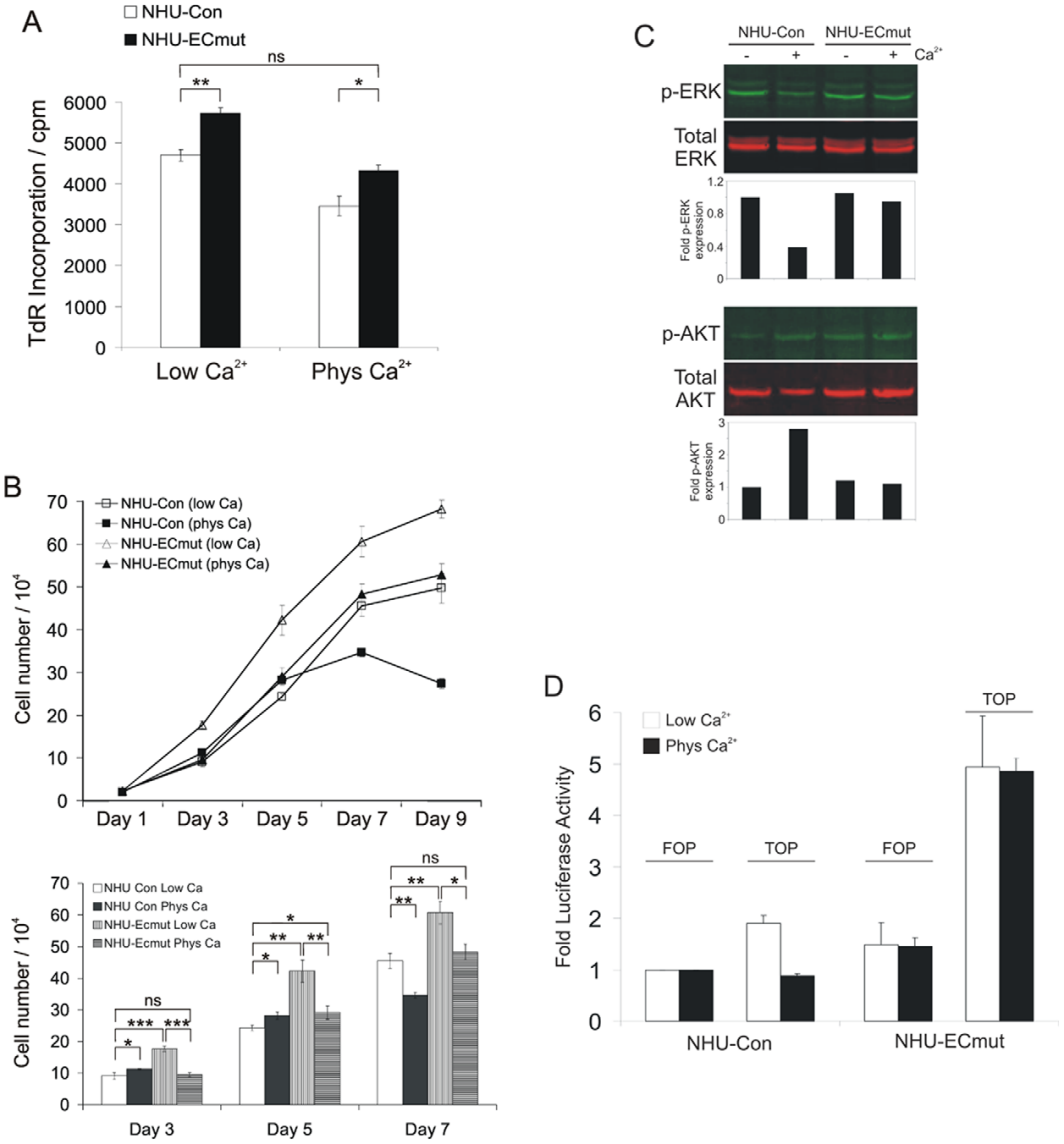
Loss of E-cadherin function enhances NHU cell proliferation and activates the EGFR/ERK and β-catenin/TCF signalling pathways. (A) NHU-Con and NHU-ECmut cells were seeded in 96-well plates and cultured as above. [^3^H]-thymidine (TdR) precursor was added 24 hours later and 16 hours post-pulsing, cells were harvested and TdR uptake measured by scintillation spectrometry. Data represent mean of cpm counts (±S.E.M.) for 12 replicate wells. ns, non-significant; *, P<0.05; **, P<0.01. Of note, NHU-Con cells showed higher proliferation levels in low calcium compared to physiologic calcium conditions, hence there was no apparent calcium-mediated enhancement in growth described above. This is because [^3^H]-thymidine incorporation studies assessed proliferation in NHU cultures that were at >50% confluence, thus missing the initial phase where cells exhibit calcium-mediated increased growth at low-density. (B) NHU-Con and NHU-ECmut cells were cultured in medium containing 0.09 mM (low Ca) or 2.0 mM (phys Ca) [Ca^2+^] and proliferation assessed by cell counting as in Figure 1A. Each data point represents the mean (±S.E.M.) of 3 replicates and results are representative of at least two independent experiments. Results for days 3, 5 and 7 are also presented in the form of bar graphs (lower panel) for the purpose of statistical analysis and clarity. ns, non-significant; *, P<0.05; **, P<0.01; ***, P<0.001. (C) NHU-Con and NHU-ECmut cells were cultured in medium containing low (-Ca^2+^) and physiological (+Ca^2+^) calcium levels for 24 hours and protein lysates were prepared. Expression of phospho-ERK (p-ERK) and -AKT (p-AKT) as well as total ERK and AKT was determined using primary and secondary antibodies described in Figures 1C and 2A. Densitometry was performed to determine fold induction of p-ERK and p-AKT expression following normalisation against total ERK and total AKT, respectively. Bar graphs represent results relative to the expression level for NHU-Con in low calcium (-Ca^2+^). (D) NHU-Con and NHU-ECmut cells were transfected with the TCF/LEF firefly luciferase reporter TOPflash or the FOPflash control plasmid, alongside the Renilla luciferase expression vector pRL-tk. Four hours post-transfection, culture supernatants were adjusted for the desired level of calcium concentration, i.e. 0.09 mM (Low Ca^2+^) or 2.0 mM (Phys Ca^2+^). Cell lysates were prepared and reporter activity assessed in a 96-well format using the Dual-Luciferase Reporter Assay on a microplate reader for pair-wise detection of luminescence activity for each reporter. Firefly luciferase values were normalised against those of Renilla luciferase and were then expressed as fold activity with respect to that obtained for FOPflash in NHU-Con cells cultured in low or physiological calcium. Bars represent mean fold Firefly luciferase activity (±S.E.M.) for 6 replicate samples.

Following retrovirus transduction and antibiotic selection, NHU-ECmut cells exhibited a consistently higher proliferation rate than the isogenic controls. This was noticeable during routine maintenance of the cultures and was confirmed quantitatively by [^3^H]-thymidine incorporation assays. These experiments showed that NHU-ECmut cells displayed consistently higher proliferation rates than NHU-Con cells in both low and physiological calcium conditions (Figure 4A). Population growth curves were also constructed over a period of 9 days. NHU-Con showed similar growth characteristics to non-transduced NHU cells, with a higher growth rate at lower density when cultured in physiological calcium (Figure 4B). In comparison to the control cells, the growth rate of NHU-ECmut was elevated in low calcium conditions and NHU-ECmut cells maintained in physiological calcium showed virtually identical growth rates to NHU-Con cells cultured in low calcium (Figure 4B); this was in agreement with the results from [^3^H]-thymidine incorporation assays (Figure 4A). Therefore, expression of mutant E-cadherin effectively overcame the inhibition of growth seen in 2 mM calcium in NHU and NHU-Con cell cultures.

We then examined the effect of abrogation of E-cadherin function on the activity of the EGFR/ERK and PI3-K/AKT pathways. The addition of physiological calcium to NHU-Con cell cultures resulted in reduced phospho-ERK levels by almost 3-fold (Figure 4C) as expected. By contrast, in NHU-ECmut cells there was little (if any) reduction in ERK phosphorylation observed (Figure 4C). At the same time, despite a substantial increase in phosphor-AKT (on Ser-473) following calcium-switching in NHU-Con cell cultures, there was no such response in NHU-ECmut cultures (Figure 4C). These findings account for the observed effect of calcium-mediated contacts in regulating growth-promoting pathways and provide direct evidence for E-cadherin-mediated modulation of EGFR/ERK and PI3-K/AKT signalling.

Our observation of EGFR/ERK de-repression following E-cadherin loss-of-function implies that elevated phospho-ERK activity may account, at least in part, for the increased proliferation rate of E-cadherin-disabled cells. However, the ability of NHU-ECmut cells to display consistently higher growth rates irrespective of extracellular calcium levels (Figure 4B) was suggestive of potential involvement of additional signalling pathways in driving cell proliferation. Another signalling candidate for promoting an increased growth rate was β-catenin, as immunofluorescence microscopy experiments revealed nuclear β-catenin localisation both in NHU cells maintained in low calcium (Figure 3B) and in NHU-ECmut cells maintained in physiological calcium (Figure 3C). β-catenin is a multifunctional protein that, upon activation, can act as a cofactor for the TCF/LEF transcription factor complex to induce or enhance cell proliferation [4]. We used a reporter assay to determine whether nuclear β-catenin was active and able to trigger TCF transcriptional activity in NHU-Con and NHU-ECmut cells. Like NHU cells (not shown), NHU-Con cells showed some β-catenin/TCF activity, which was diminished in the presence of physiological calcium (Figure 4D). By contrast, NHU-ECmut cells showed high levels of β-catenin/TCF activity, irrespective of the extracellular calcium concentration (Figure 4D). Therefore, collectively, our studies show that functional inactivation of E-cadherin in NHU cells de-repressed EGFR/ERK signalling and enhanced β-catenin/TCF transcriptional activity by several-fold.

### Down-regulation of β-catenin by RNA interference mimics E-cadherin engagement and influences PI3-K/AKT activity and NHU cell proliferation

As shown above, whilst inducing PI3-K/AKT activity (Figure 2), E-cadherin recruits β-catenin to the points of cell contact (Figure 3B) and down-regulates β-catenin/TCF activity (Figure 4D), whereas loss of E-cadherin function abolishes PI3-K/AKT induction (Figure 4C) and confers constitutive activation of β-catenin (Figure 4B). Collectively, these observations suggest that E-cadherin-mediated sequestration of active β-catenin to the cell surface coincides with induction of PI3-K/AKT activity. We therefore next addressed the possibility that engineered loss of active β-catenin may mimic the effects of E-cadherin engagement. We produced NHU cell derivatives that were transduced with a retrovirus expressing a β-catenin-specific short-hairpin RNA (shRNA) adapted from a reported siRNA sequence [25].

**Figure 5:**
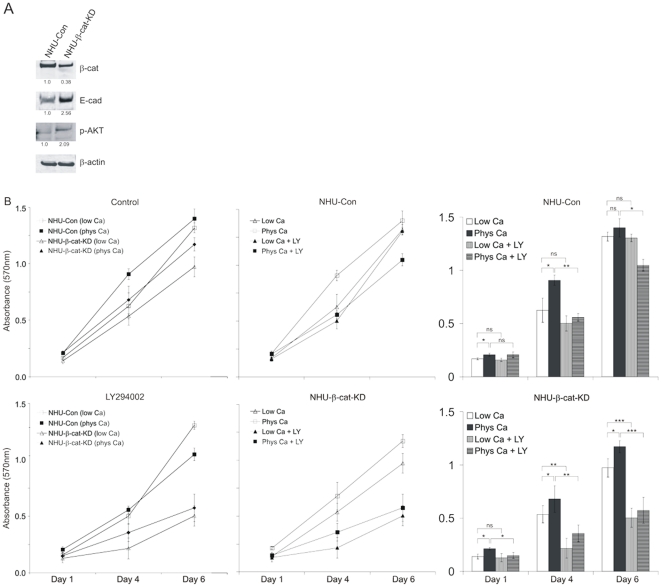
Loss of β-catenin by shRNA-mediated knock-down mimics E-cadherin engagement. (A) NHU cells expressing β-catenin shRNA (NHU-β-cat-KD) and their isogenic controls expressing scramble shRNA control (NHU-Con) were established by retrovirus transduction and the efficiency of the shRNA to down-regulate β-catenin was assessed by immunoblotting. Lysates from NHU-Con and NHU-β-cat-KD cells maintained in low calcium were probed for expression of active β-catenin (β-cat), E-cadherin (E-cad), phospho-AKT at S437 (p-AKT) and β-actin using mouse antibodies (β-cat, E-cad and β-actin) and rabbit antiserum (p-AKT), followed by goat anti-mouse antibody conjugated with Alexa Fluor 680 or goat anti-rabbit antibody conjugated with IRDye 800 as appropriate. Densitometry was performed to determine differences in protein expression with respect to β-actin and fold differences following normalisation are indicated. (B) NHU-Con and NHU-β-cat-KD cell lines were seeded into 96-well plates and cultured for a period of 6 days in medium containing either 0.09 mM (Low Ca) or 2.0 mM (Phys Ca) calcium concentration and in the absence (Control) or presence of 5 µM of PI3-K inhibitor LY294002. Proliferation was determined on the basis of cell biomass using the MTT assay and results are presented in upper and lower left panels. Data points represent mean absorbance values for 6 replicate wells (±S.E.M.). To allow easier comparison between inhibitor-treated (+ LY) and non-treated cells, graphs for each cell line are also presented (upper and lower middle panels). In addition, results for all data points are shown in the form of bar graphs (lower panel) for the purpose of statistical analysis. ns, non-significant; *, P<0.05; **, P<0.01; ***, P<0.001.

Expression of active β-catenin was significantly decreased in β-catenin shRNA-expressing (NHU-β-cat-KD) cells when compared to control (NHU-Con) cells expressing control shRNA, as shown by immunoblotting experiments (Figure 5A). The shRNA-mediated β-catenin knock-down resulted in the induction of phospho-AKT and this was accompanied by an almost three-fold increase in E-cadherin expression (Figure 5A). This suggested that forced down-regulation of active β-catenin allowed induction of PI3-K/AKT activity. To confirm the functional involvement of the induced phospho-AKT, we assessed population growth for NHU-Con and NHU-β-cat-KD cell cultures in 0.09 mM or 2 mM calcium in the presence of the LY294002 inhibitor over a 6 day time-course (Figure 5B). As in NHU cells, the increased proliferation rate characteristic of cultures grown in physiological calcium was attenuated by LY294002 treatment in NHU-Con cells, whereas the inhibitor had little effect on the exponential growth rate in low calcium conditions. However, NHU-β-cat-KD cell growth in physiological calcium was significantly reduced in comparison to cultures of NHU-Con cells (Figure 5B), indicating that β-catenin knock-down rendered NHU- β-cat-KD cells very sensitive to the LY294002 inhibitor and confirming the importance of PI3-K/AKT signalling in conditions that mimic E-cadherin engagement.

## Discussion

Our study indicates a critical role for E-cadherin in monitoring the “quality” of cell:cell interactions and regulating density-dependent cell growth via the modulation of different growth promoting pathways. Thus, E-cadherin has the capacity to simultaneously regulate signalling through EGFR/ERK, PI3-K/AKT and β-catenin/TCF pathways, with direct, context-specific consequences for individual cell proliferation within a population. In addition to demonstrating a central role for E-cadherin in regulating epithelial tissue homeostasis and regeneration, it also provides insight into the downstream sequelae of E-cadherin loss in cancer.

The well-recognised property of E-cadherin as a metastasis suppressor is generally attributed to its function in mediating adherens junctions in the maintenance of tissue integrity. Although it is also accepted that E-cadherin has a modulatory role in cell proliferation and migration, its specific contribution has remained more equivocal. One problem of interpretation is that reintroduction of E-cadherin expression into a malignant cell line tends to reveal idiospecific effects, as it is unlikely to restore all normal functions of E-cadherin against a background of multiple genetic/epigenetic alterations. Pece and co-workers demonstrated E-cadherin-dependent, ligand-independent activation of EGFR and downstream MAPK pathways in the HaCaT cell line [7]. This was consistent with E-cadherin providing a proliferation signal in keratinocytes [8], [26], or rescue from anoikis in squamous cell carcinoma [27]. However, in other cell types, such as mammary epithelial cells [10], [28], [29], as well as melanoma and breast cancer cell lines [11], E-cadherin-mediated cell-cell contacts have been shown to suppress proliferation signals that are driven by RTKs, particularly from the EGFR pathway. These contradictory findings prompted us to study the precise influence of E-cadherin on downstream growth-promoting cell signalling pathways in a normal epithelial cell system.

At low density, NHU cells grown in physiological calcium exhibited transiently-higher growth rates than cultures grown in low calcium, which was attributable to activation of the PI3K/AKT pathway. That this was the result of functional E-cadherin cell contacts, rather than being attributable to the increase in calcium alone, was indicated by the confinement of AKT activation to juxtaposed NHU cells and the loss of the effect in E-cadherin-null cultures. These observations are supported by the findings of Liu and co-workers, who showed that E-cadherin-mediated contacts were responsible for promoting proliferation in mammary epithelial cells cultured at low densities [30], as well as a study reporting that induction of cell proliferation by cell:cell contacts in endothelial and smooth muscle cells was associated with PI3K/AKT activation [31].

Our study was originally instigated on the basis of in vitro/in virtuo comparison with an agent-based computational model of epithelial cell interactions [17], [18], [19]. The most recent version of the model incorporates a mathematical model of juxtacrine EGFR/ERK activation to explore the importance of cell-cell contact in regulating ERK activity and NHU cell proliferation [20]. The modelling results predicted that transient cell contacts occurring in low calcium conditions would result in rapid but transient ERK activation. Simulated “virtual immunoblotting” data for a population of cells suggested that transient contacts resulted in a high overall phospho-ERK signal. By comparison, slowly-formed contacts stabilised by the formation of E-cadherin junctions in physiological calcium conditions resulted in sustained ERK activation, but the overall amount of phospho-ERK for the total population was less than for cultures maintained in low calcium concentrations [20]. The present experimental work confirmed the model's prediction that in physiological calcium conditions, the amount of ERK phosphorylation for the population is less than for an equivalent culture grown in low calcium conditions. By extending our study to the AKT pathway, we have also now provided an explanation for the increase in proliferation rate observed previously in NHU cell cultures grown in physiological calcium [17].

As E-cadherin contacts suppress EGFR/ERK signalling in urothelial cells, the implication is that E-cadherin loss may directly contribute to the activation of RTKs frequently reported in carcinomas [32], including those of the bladder ([33] and references therein). Engineering NHU cells with functionally-inactivated E-cadherin enabled us to specifically probe the importance of E-cadherin-associated cell contacts in cell proliferation. Expression of the dominant-negative H-2K^d^-E-cad mutant down-regulated endogenous E-cadherin by blocking its localisation to sites of cell-cell contact and abrogating the recruitment of α-catenin and β-catenin. The H-2K^d^-E-cad chimera interacted with endogenous protein to recruit it to perinuclear, Golgi/late-endosome compartments, reminiscent of the situation with normal keratinocytes [22], but not immortalised HaCaT cells, where surface localisation was reported [34]. Inactivation of E-cadherin abolished calcium-mediated growth restraints, allowed de-repression of EGFR/ERK, diminished the influence of PI3-K/AKT in NHU cell proliferation at low density and “released” β-catenin to permit its re-localisation to the nucleus. The β-catenin knock-down experiments revealed a mutually-exclusive relationship between PI3-K and β-catenin signalling, as well as demonstrating that active β-catenin has a repressor role on E-cadherin expression. As a result of the loss of functional E-cadherin, the de-repression of EGFR/ERK and constitutive activation of β-catenin/TCF signalling (discussed below) allowed NHU cells to resume their high proliferative potential even in physiological calcium conditions.

Unlike previous studies that have suggested no constitutive Wnt/β-catenin signalling in NHU cells [35], we believe that our work provides the first evidence for an activated Wnt signalling pathway in urothelial cells, as assessed by TCF/LEF reporter assay in conditions where E-cadherin signalling was suppressed (low calcium medium or expression of the H-2K^d^-E-cad chimera). Various reports give credence to a role for Wnt signalling in bladder cancer, including epigenetic loss of Wnt inhibitory components, such as WIF-1, DKK subunits and secreted frizzled-related proteins (sFRP) [36], [37], hypermethylation of the APC locus [38] and loss of E-cadherin expression via CDH1 locus hypermethylation or mutation [39]. This emergent evidence in combination with our findings indicates that the down-regulation of E-cadherin is permissive for Wnt signalling in urothelial cells.

It is well-established that E-cadherin plays a critical role in epithelial carcinogenesis due to its importance in regulating cell-cell adhesion [40]. E-cadherin expression is frequently lost in aggressive epithelial cancers, including those of the bladder, prostate, lung and pancreas [41]. In the case of bladder cancer, loss of E-cadherin expression is a common event [42], which occurs in advanced tumours as a result of CDH1 locus hypermethylation or mutation [39] and there is evidence for a strong association between hypermethylation of the E-cadherin locus with high disease-progression risk [43]. Because restoration of its expression often leads to growth-retardation, reduction of invasive properties [44] and/or reversion of the tumour phenotype from malignant to benign [45], [46], E-cadherin has been generally termed a metastasis suppressor [47]. Sensitivity to the EGFR inhibitor, cetuximab, has been shown to require intact E-cadherin expression and silencing of E-cadherin by RNA interference was reported to reduce responsiveness to EGFR inhibition in previously sensitive bladder cancer cell lines [48]. Although no biological evidence was provided, the authors proposed that the requirement for E-cadherin to achieve increased growth inhibition by cetuximab might indicate a mechanism of E-cadherin-mediated EGFR activation. We believe that our observation of E-cadherin-mediated down-regulation of EGFR/ERK is not contradictory and may provide a mechanistic explanation for these findings. Our results imply that a reduced response to EGFR inhibitor by E-cadherin-negative cells [48] may, at least in part, be due to high amounts of EGFR-driven ERK activation resulting from loss of E-cadherin function, thus reducing the efficacy of cetuximab treatment. More importantly, loss of E-cadherin may result in activation of additional growth-promoting signalling pathways normally attenuated by E-cadherin-mediated contacts, such as activation of Wnt and β-catenin/TCF signalling; such signals would not be attenuated by cetuximab-mediated EGFR inhibition.

In summary, following a functional inactivation approach our studies provide, for the first time, molecular evidence that E-cadherin engagement can dictate the activity of several mitogenic signalling pathways, as it differentially regulates EGFR/ERK, PI3-K/AKT and β-catenin/TCF. Studies using normal epithelial cell cultures [49], including our own, clearly suggest that E-cadherin loss-of-function may provide a growth advantage. This indicates that its role in carcinogenesis is not limited to metastatic spread, but that impairment of E-cadherin may provide a growth advantage at an earlier stage – such as in non-malignant, low-grade papillary neoplasms of the bladder [50], early-stage carcinomas of the cervix [51], or in tumours where E-cadherin protein is expressed heterogeneously [50], [52]. The understanding of homeostatic regulation of normal epithelial tissue regeneration could have profound implications for developing therapies to target key growth promoting pathways.

## Materials and Methods

### Ethics Statement

NHU cell lines were established from urological specimens obtained with written informed patient consent and approval from the Leeds (East) and York NHS Research Ethics Committees.

### Cell culture

NHU cell lines of finite lifespan were established and maintained in keratinocyte serum-free medium containing bovine pituitary extract, recombinant EGF and supplemented with cholera toxin (KSFMc), using methods that have been described in detail elsewhere [12], [14]. NHU cells were used for retrovirus transduction at passages 1 or 2 and subsequent experiments were performed between passages 2-6. Retrovirus packaging PT67 fibroblasts (BD Biosciences, Oxford, UK) were maintained in DR medium, consisting of a 1∶1 (v:v) mixture of DMEM and RPMI 1640 (Invitrogen, Paisley, UK), containing 10% fetal bovine serum (FBS, Harlan Sera-Lab, Loughborough, UK).

### Preparation of retroviral vectors and retrovirus-producing cell lines

The dominant-negative H-2Kd-E-cad mutant [26] was the gift of Professor Fiona Watt (CRUK Research Institute, Cambridge, UK). H-2K^d^-E-cad cDNA was sub-cloned from pBabe-puro into the XhoI site of the pLXSP retroviral plasmid [53]. Recombinant vectors pLXSP-ECmut and pLXSP-ECrev were derived, containing the H-2K^d^-E-cad fragment in the forward and reverse (non-sense) orientations, respectively (pLXSP-ECrev was used as a specificity control). For the preparation of short hairpin RNA (shRNA) to target β-catenin, an siRNA previously reported [25] was used and adapted for delivery using the pSIREN-RetroQ retroviral vector system. Oligonucleotide primers were prepared and cloned into pSIREN-RetroQ according to the manufacturer's instructions (Clontech, BD Biosciences), giving rise to vector pSIR-β-cat. The pSIR-Con vector, which contained a scrambled shRNA sequence, was used alongside as negative control.

### Retroviral transduction of NHU cells

Retroviral transductions were performed as described previously [53], [54]. PT67 fibroblasts were transfected with pLXSP-ECrev, pLXSP-ECmut, pSIR-Con and pSIR-β-cat vectors using Effectene™ (Qiagen, Crawley, UK). Stable retrovirus-producing lines PT67-ECrev, PT67-ECmut, PT67-Con-sir and PT67-β-cat-sir were established by selection in culture medium containing 4 µg/mL puromycin (Autogen Bioclear UK Ltd, Calne, UK). Conditioned media from stable retrovirus producers were 0.45 µm membrane-filtered, supplemented with 8 µg/mL polybrene (SigmaAldrich, Poole, UK) and used to infect NHU cell cultures for 5–6 hours. The medium was replaced with KSFMc and two days post-transduction, NHU cells were subjected to antibiotic selection using 1.5 µg/mL puromycin. 2–3 independent NHU lines were each transduced with PT67-ECrev, PT67-ECmut, pSIR-Con and pSIR-β-cat virions resulting in the derivation of isogenic NHU-Con and NHU-ECmut sub-lines as well as NHU-Con and NHU-β-cat-KD sub-lines.

### Cell proliferation assays

NHU cells and virally-transduced derivatives were cultured in KSFMc containing low (0.09 mM) or physiological (2.0 mM) Ca^2+^ and proliferation was assessed by cell counting, by colorimetric assay with thiazolyl blue tetrazolium (MTT; SigmaAldrich) and by [^3^H]-thymidine incorporation. For counting, cells were seeded in 6-well plates at 2×10^4^ cells/well and counts performed for a period of 9 days. For MTT assays, cells were seeded in 96-well plates at 4×10^3^/well and proliferation was assessed over the indicated time-course in the presence or absence of 5 µM LY294002. Following treatment with MTT reagent (500 µg/mL) (SigmaAldrich), absorbance at 570 nm was measured. For [^3^H]-thymidine (TdR) incorporation [53], cells were seeded in 96-well plates at 2×10^4^/well; TdR precursor (GE Healthcare, Bucks, UK) was added 24 hours later at 0.5 µCi/well and 16 hours post-pulsing, cells were harvested onto filter-mats and TdR uptake was measured by scintillation spectrometry.

### Flow cytometry

H-2K^d^ expression was analysed using a FITC-conjugated antibody and an isotype control recommended by the manufacturer (BD Biosciences). Using previously described methods [54], [55], NHU cells were labelled with these antibodies and analysed by flow cytometry on the FL-1 (FITC) channel. A minimum of 10,000 cells were acquired on a CyAn™ instrument and protein expression was analysed using Summit® software (DakoCytomation, Ely, UK).

### Immunoblotting

Cell lysates were prepared, resolved on NuPage® 4–12% bis-Tris acrylamide gels in MES or MOPS running buffer (Invitrogen) and transferred onto 0.45 µm PVDF membranes (GE Healthcare, Bucks, UK) as previously [53], [56]. Affinity-purified polyclonal antibodies recognising EGFR (cat#2232), phospho-ERK (cat#9101) and phospho-AKT S473 (cat#9271s) were from Cell Signalling (Autogen Bioclear). Monoclonal antibodies against total-ERK (clone#16) and total-AKT (clone#7) were from BD Biosciences, β-actin (clone#AC-15) was from SigmaAldrich and E-cadherin (mouse monoclonal clone #HECD-1) was from R&D systems (Abingdon, UK). The 8E7 monoclonal antibody recognising active, unphosphorylated β-catenin was a kind gift of Prof Hans Clevers (Hubrecht Institute, UMC, Utrecht, Netherlands). Membranes were probed with primary antibodies followed by goat anti-rabbit immunoglobulins conjugated to IRDye® 800 (Tebu-bio, Peterborough, UK) or goat anti-mouse antibody conjugated to Alexa Fluor® 680 (Invitrogen). Immunolabelling was visualised and densitometry performed using an Odyssey system (LiCor, Cambridge, UK).

### Immunofluorescence microscopy

NHU cell cultures were fixed with 10% formalin in PBS and permeabilised in PBS containing 0.1% Triton-X and 10% goat serum (SigmaAldrich). Antibodies used were: α- and β-catenin (rabbit, cat#C-2081 and cat#C-2206, respectively; SigmaAldrich). Antibodies against H-2K^d^, EGFR, E-cadherin, total/phospho-ERK and total/phospho-AKT were as above. Following labelling with primary antibody, cells were washed and incubated with goat anti-mouse or anti-rabbit immunoglobulins conjugated with either Alexa Fluor® 488 or 594 (Invitrogen). Cell nuclei were stained using 0.1 µg/mL Hoechst 33258 (SigmaAldrich). Labelled preparations were observed by epifluorescence on an Olympus BX60 microscope.

### Luciferase reporter assays

Cells were seeded in 24-well plates at 5×104/well and transfected as previously [54] using Effectene™ with 0.5 µg of either TOPflash (luciferase construct containing tandem repeats of the minimal TCF promoter) or FOPflash (negative reporter control construct that contains mutant TCF binding sites) plasmid (Millipore, Watford, UK). Reporter plasmids were transfected alongside 0.1 µg of Renilla luciferase expression vector pRL-tk as a transfection control and for normalisation of luciferase activity. Cell lysates were prepared and reporter activity assessed in a 96-well format by the Dual-Luciferase™ Reporter Assay according to the manufacturer's instructions (Promega, Southampton, UK) on a POLARstar OPTIMA microplate reader (BMG Labtech, Aylesbury, UK).

### Statistics

Means and standard errors from the mean (S.E.M.) were used as descriptive statistics. Unless otherwise stated, Student's t-test was used for evaluation of statistical significance.
